# Perceptions of medical students towards the practice of professionalism at the Arabian Gulf University

**DOI:** 10.1186/s12909-020-02464-z

**Published:** 2021-01-08

**Authors:** Haifa Mohammed Saleh Al Gahtani, Haitham Ali Jahrami, Henry J. Silverman

**Affiliations:** 1 Department of Psychiatry, Arabian Gulf University, Manama, Kingdom of Bahrain; 2 Department of Medicine, University of Maryland Baltimore, Baltimore, MD 21201 USA

**Keywords:** Professionalism, Medical students, Teaching professionalism, Behaviors

## Abstract

**Background:**

To enhance the development of a curriculum in professionalism for medical students, the aim of this research was to evaluate medical students’ responses regarding professionalism teaching and behaviors in their clinical experience at the Arabian Gulf University (AGU).

**Methods:**

A retrospective cross-sectional, questionnaire-based study involving Year 5 medical students at the AGU. We used a “climate of professionalism” survey that consisted of two parts. The first part asked students to rate their perceptions of the frequency of professionalism practices of their peers (medical students), residents, and faculty. The response choices included: “mostly”, “sometimes”, and “rarely”. The second part asked the students to assess their perceptions of the professionalism teaching and behaviors of the faculty. The response choices included: “mostly”, “sometimes”, and “rarely”. We calculated an overall score for the responses in both parts of the questionnaire by assigning 3, 2, and 1 points to the response choices, respectively. We also calculated subscale scores reflecting different professionalism constructs. We used descriptive statistics and a one-way Analysis of Variance (ANOVA) followed by multiple testing comparisons with Bonferroni correction to examine pairwise comparisons. A *p* < 0.05 was considered statistically significant.

**Results:**

The mean total scores of participants’ ratings of professional behaviors of medical students, residents, and faculty for each academic year were approximately 60% of the total maximum score. The mean total scores of participants’ rating of faculty’s teaching and modeling behaviors concerning professionalism were approximately 58% of the maximum score. Compared with similar studies performed in the Arab Region, ratings regarding professional teaching and modeling of professionalism were lower.

**Conclusion:**

We recommend the further evaluation of professionalism teaching and behaviors at the AGU and further discussions regarding curriculum reform.

**Supplementary Information:**

The online version contains supplementary material available at 10.1186/s12909-020-02464-z.

## Background

There has been an increased emphasis on teaching medical professionalism in the last decade due to public dissatisfaction with the performance of the medical profession [1]. As patients entrust their health to health care providers, graduates will find professionalism an essential quality to complement their biomedical knowledge and clinical skills. Studies have shown that medical professionalism is associated with improvement in patient-physician relationships, patient and professional satisfaction, and healthcare outcomes [2].

Professionalism refers to a collection of values, attitudes, behaviors, and relationships that act as the foundation of the healthcare profession’s contract with society [3]. The concept of medical professionalism has evolved from the earliest teachings of Hippocrates, [4] who described physician behaviors expected of them and Alī al-Ruhāwī, [5] who defined the characteristics of a virtuous physician, to the more recent charter of medical professionalism that outlined and discussed discrete principles [6]. The importance of professionalism to medical students represents a commitment to professional skills and attitudes that they will acquire reliably and lead to public trust in the medical profession.

An established framework that lists the attributes of medical professionalism includes the American.

Board of Internal Medicine (ABIM), which consists of six domains of professionalism; altruism, accountability, excellence, duty, honor and integrity and respect for others [7]. This framework of medical professionalism has been considered to be applicable in other countries, [8–10] including the Arab Region [11].

Although there have been differences of opinions in regards to how to teach professionalism, [12, 13] another issue involves efforts to measure professionalism in the clinical environment [14, 15]. Several surveys have assessed the climate of the clinical environment, thus focusing on aspects of the environment related to professionalism, such as teamwork, warmth, respect, and social responsibility [16–18]. Alternatively, others have performed surveys asking individuals in medical training to assess the professional behaviors of their peers and instructors, as the teaching of professionalism depends heavily on role-modeling [15, 19, 20].

Recognizing the importance of professionalism, the College of Medicine and Medical Sciences at the Arabian Gulf University (AGU) in Bahrain incorporated the teaching of professionalism into its medical school curriculum through the medium of a yearly 2-day workshop. Students participating in the workshop were asked to complete a “climate of professionalism” questionnaire that we adapted from the one developed originally by Quaintes et al., [15] and tested in other studies [21]. As learning objectives regarding the teaching of professionalism to medical students lack consensus, [22] we decided to analyze the results of this “climate of professionalism” questionnaire to inform the teaching of professionalism in subsequent years of the workshop at AGU. Accordingly, the aim of this research was for us to systematically evaluate the medical students’ responses regarding professional teaching and behaviors present in the clinical areas at AGU.

## Methodology

### Study design

A retrospective cross-sectional, questionnaire-based study. We performed all methods in accordance with relevant guidelines and regulations.

### Participants

Year 5 students who attended the Professionalism and Ethics workshop for the academic years 2016–2017. 2017–2018, 2018–2019, and 2019–2020.

### Setting - the teaching of professionalism

Studying medicine at the Arabian Gulf University (AGU) spans over six years where students pass through a pre-clinical phase that includes years 1–4 and a clinical phase that includes years 5 and 6. In the preclinical phase, students learn about different components of professionalism as part of their weekly problem-based learning (PBL) tutorials. In addition, they attend a half/day professionalism course in the second year. In the clinical phase, an additional mandatory two-day workshop was added to the curriculum in 2016 where students, residents and faculty discuss the main principles and values of professionalism. This workshop employed active learning techniques consisting of “think-pair-share”, videos, polling, and small group discussions of common clinical cases faced in their daily practice, which were then followed by large group discussions.

### Questionnaire

We adapted the “climate of professionalism” questionnaire from Quaintes and colleagues [15]. The questionnaire consists of two parts. The first part assesses the students’ perceptions of the frequency of professionalism practices during their clinical years. This part consists of 11 different professional and unprofessional behaviors in the clinical environment for three different target groups: medical students, residents, and faculty. The response choices included: “mostly”, “sometimes”, and “rarely”. Thus, there was a total of 36 items. Examples of these items include the following: students/residents/faculty “advocate for the well-being of patients, students, colleagues, the community and/or the medical profession” and students/residents/faculty “complain about professional obligations.”

The second part elicit students’ perceptions regarding the frequency with which their professors/supervisor/attending physicians taught and modeled ten different professional behaviors over the past year. Response choices included: “mostly”, “sometimes”, and “rarely”. Thus, there was a total of 30 items. Both parts of the questionnaire are available as supplementary files (1).

### Distribution of questionnaires

We distributed the self-administered questionnaire to the medical students via the Moodle online learning platform after they attended the yearly two-day workshop in Professionalism. Responses were collected anonymously.

### Statistical analysis

From the items in Part I of the questionnaire, we calculated a total “professionalism behavior” score for each group (medical students, resident, and faculty) by assigning points to the following responses: three points to “mostly”, two points to “sometimes”, and one point to “rarely”. Negatively worded professionalism behaviors were reverse scored so that high scores reflected more positive behaviors. The range of these scores for each group is 11–33. Higher scores indicate higher perceptions of professional behaviors.

From the 11 items in Part I of the questionnaire, we also calculated subscale scores reflecting different professionalism constructs adapted from the ABIM definition of professionalism [7]. These include the dimensions of a) Respect/Caring/Compassion/Altruism, b) Honesty/integrity,

c) Accountability/Responsibility, and d) Duty/Service/Excellence. The professionalism behaviors associated with each of these subscales are revealed in the Table 1. This table also shows the range of points assigned to each subscale, which was based on the above-mentioned rubric that we used to calculate the total “professionalism behavior” score.

**Table 1:** Professionalism behaviors associated with each subscale

**Subscale 1 Respect/Caring/Compassion/Altruism (score range: 3–9)**	
Show disrespect to patients, students, faculty, staff, or other healthcare personnel.	
Show respect and compassion toward patients, students, faculty, staff or other healthcare personnel.	
Advocate for the well-being of patients, students, colleagues, the community and/or the medical profession.	
**Subscale 2 Honesty/integrity (score range: 3–9)**	
Make selves look good at the expense of others.	
Complain about professional obligations.	
Lie to patients, professors, colleagues/peers or in the medical record.	
**Subscale 3 Accountability/responsibility (score range: 2–6)**	
At times hide their medical mistakes from their colleagues and the patients.	
Ignore the unprofessional behavior of others.	
**Subscale 4 Duty/Service/Excellence/Altruism (score range: 3–9)**	
Exceed expectations inpatient care, class, conferences, and/or rounds.	
Finish their work and help others finish theirs.	

We performed a Cronbach alpha (coefficient alpha) on the entire scale to check the internal consistency. Our analysis showed an overall Cronbach alpha of 0.60, suggesting that the questionnaire is sufficiently reliable for further analyses. Cronbach alpha also revealed that no item if deleted would improve the overall consistency.

For the 10 items in Part II of the questionnaire, we calculated a total score for “faculty’s teaching and modeling professional behaviors” by assigning points to the following responses: three points to “mostly”, two points to “sometimes”, and one point to “rarely”. The range of scores is 10–30.

We analyzed the data by using the Statistical Package for Social Sciences (SPSS) - version 26. We used descriptive analyses for all the data. We used a one-way Analysis of Variance (ANOVA) to test for mean differences between the three groups and also between the different academic years, followed by multiple testing comparisons with Bonferroni correction to examine pairwise comparisons. Statistical significance was at the level of *p* < 0.05.

## Results

From the four academic years, a total of 520 students completed the questionnaires; 120 students were from the academic 2016/2017; 135 students from the academic year 2017/2018, 149 students from the academic year 2018/2019, and 116 were from the academic year 2019/2020.

Table 2 shows the students’ frequency rating with which they observed each of the professional behaviors of medial students, residents, and faculty in a clinical environment. The ratings represent the mean cumulative percentages of all four academic years. Professional behaviors that the medical students rated > 50% for the “mostly” observed category for all three groups included: “Show respect and compassion toward patients, students, faculty, staff or other healthcare personnel” and “show respect and compassion toward patients, students, faculty, staff or other healthcare personnel”.

**Table 2:** Participants’ rating of the frequency they observed members in each group exhibiting each behavior during the past year. Ratings represents cumulative data for all four academic years Total n (all four years) =520

Professionalism Behaviors	Medical Students			Residents			Faculty		
	Mostly %	Sometimes %	Rarely %	Mostly %	Sometimes %	Rarely %	Mostly %	Sometimes %	Rarely %
1. Show disrespect to patients, students, faculty, staff or other healthcare personnel.	67	20	12	44	47	9	49	37	14
2. Advocate for the well-being of patients, students, colleagues, the community and the medical profession.	44	48	8	49	43	8	48	35	17
3. Make selves look good at the expense of others.	29	49	23	32	53	15	36	44	20
4.Exceed expectations in patient care, class, conferences and/or rounds.	11	67	22	27	60	13	30	61	10
5. Finish their work and help others finish theirs.	33	47	20	36	46	18	25	53	22
6. Complain about professional obligations.	24	49	27	34	56	10	41	44	15
7. Lie to patients, professors, colleagues/peers or in the medical record.	48	39	12	57	36	7	59	31	10
8. Show respect and compassion toward patients, students, faculty, staff or other healthcare personnel.	69	26	4	57	35	8	56	35	9
9. At times hide their medical mistakes from their colleagues and the patients.	38	49	13	39	48	13	34	50	16
10. Ignore the unprofessional behavior of others.	15	58	27	28	61	11	40	41	20
11. Do just enough to get by in patient care.	12	52	36	17	53	31	15	59	26

*P< 0.05

Unprofessional behaviors rated > 30% for the “mostly” observed category for all three groups included: “Show disrespect to patients, students, faculty, staff or other healthcare personnel”; “lie to patients, professors, colleagues/peers or in the medical record”, and “at times hide their medical mistakes from their colleagues and the patients”.

Table 3 shows the mean total scores of medical students’ ratings of professional behaviors of medical students, residents, and faculty for each academic year. As the maximum total score is 33, all of the mean total scores for all groups and for all four years were approximately 60% of the total maximum Professionalism score.

**Table 3:** Mean TOTAL scores of participants’ rating of professional behaviors of medical students, residents, and faculty (maximum total score = 33)

	Medical Students	Residents	Faculty	ANOVA
Year 2016–2017 *n* = 120	20.31 ± 3.26	19.38 ± 3.11	19.62 ± 3.27	0.07
Year 2017–2018 *n* = 135	20.24 ± 3.42	19.3 ± 3.21	19.39 ± 3.3	0.04
Year 2018–2019 *n* = 149	19.93 ± 3.11	18.95 ± 2.98	19.23 ± 3.12	0.02
Year 2019–2020 *n* = 116	20.18 ± 2.96	19.31 ± 3.14	19.59 ± 3.27	0.10

For the academic years 2017/2018 and 2018/2019, the medical students had mean total scores that were significantly greater than the groups comprised “residents” and “faculty”. This difference was not present for the academic years 2016/2017 and 2019/2020.

Table 4 shows the mean subscale scores representing the four different professionalism behavior constructs for each of the groups and for each of the four academic years. For the subscale Respect/Caring/Compassion/Altruism, all of the mean scores were approximately 55% of the maximum score of 6. For the subscale Honesty/integrity, all of the mean scores were approximately 65% of the maximum score of 9. For the subscale Accountability/Responsibility, all of the mean scores were approximately 65% of the maximum score of 6. Finally, for the subscale Duty/Service/Excellence, all of the mean scores were approximately 68% of the maximum score of 9.

**Table 4:** Mean subscale scores of participants’ rating of professional behaviors of medical students, residents, and faculty

	Medical Students	Residents	Faculty	ANOVA
**Year 2016–2017 (*n*** **= 120)**				
Subscale 1 Respect/Caring/Compassion/Altruism (range 3–9)	4.59 ± 1.33	4.79 ± 1.43	4.88 ± 1.51	0.285
Subscale 2 Honesty/integrity (range 3–9)	5.71 ± 1.4	5.15 ± 1.30	5.18 ± 1.34	0.002
Subscale 3 Accountability/responsibility (range 2–6)	3.88 ± 0.92	3.59 ± 1.02	3.67 ± 1.06	0.079
Subscale 4 Duty/Service/Excellence (range 3–9)	6.13 ± 1.19	5.85 ± 1.29	5.89 ± 1.16	0.152
**Year 2017–2018 (*n*** **= 135)**				
Subscale 1 Respect/Caring/Compassion/Altruism	4.48 ± 1.29	4.76 ± 1.41	4.84 ± 1.57	0.091
Subscale 2 Honesty/integrity	5.68 ± 1.41	5.07 ± 1.28	4.97 ± 1.30	0.000
Subscale 3 Accountability/responsibility	3.82 ± 0.93	3.63 ± 0.99	3.65 ± 0.99	0.206
Subscale 4 Duty/Service/Excellence	6.26 ± 1.23	5.84 ± 1.35	5.92 ± 1.19	0.015
**Year 2018–2019 (*n*** **= 149)**				
Subscale 1 Respect/Caring/Compassion/Altruism	4.40 ± 1.24	4.66 ± 1.4	4.84 ± 1.53	0.026
Subscale 2 Honesty/integrity	5.56 ± 1.35	5.00 ± 1.24	5.04 ± 1.28	0.000
Subscale 3 Accountability/responsibility	3.81 ± 0.93	3.51 ± 0.96	3.51 ± 1.00	0.010
Subscale 4 Duty/Service/Excellence	6.17 ± 1.09	5.79 ± 1.21	5.84 ± 1.08	0.007
**Year 2019–2020 (*n*** **= 116)**				
Subscale 1 Respect/Caring/Compassion/Altruism	4.34 ± 1.19	4.78 ± 1.44	4.87 ± 1.51	0.009
Subscale 2 Honesty/integrity	5.53 ± 1.31	5.13 ± 1.32	5.16 ± 1.36	0.043
Subscale 3 Accountability/responsibility	3.99 ± 0.90	3.59 ± 1.03	3.66 ± 1.06	0.005
Subscale 4 Duty/Service/Excellence	6.32 ± 1.18	5.82 ± 1.30	5.89 ± 1.16	0.003

For all four years, the medical student group had mean subscale scores for “Honesty/Integrity” that were significantly higher compared with the other two groups. For the last three academic years, the medical student group had mean subscale scores for “Duty/Service/Excellence” that were significantly higher compared with the other two groups. Finally, for the last two academic years, the medical student group had mean subscale scores for “Accountability/responsibility” that were significantly higher compared with the other two groups. In contrast, for the last two academic years, the faculty group had mean subscale scores for “Respect/Caring/Compassion/Altruism” that were significantly higher compared with the other two groups.

Table 5 shows the medical students’ perceptions of faculty’s teaching and modeling professionalism behaviors. The ratings represent the mean cumulative percentages of the combined four academic years. More than 40% of the medical students rated “mostly” the following observed teaching behaviors of faculty: “acts professionally in relating to patients, students, colleagues and staff”; teaches about professionalism”; “is a good role model of professionalism for me to emulate”; and “sets clear expectations for student’s professional behavior”. However, at least 25% of the medical students rated “rarely” the following two teaching behaviors: “after describing the way a student should relate to a patient in a difficult situation, demonstrates that behavior for students”; and “after the demonstration, asks students what they saw and solicits their comments”.

**Table 5:** Participants’ rating of faculty in regard to professionalism. Ratings represents cumulative data for all four academic years. Total *n* = 520

Professionalism Teaching Behaviors	Mostly %	Sometimes %	Rarely %
1. Acts professionally in relating to patients, students, colleagues and staff.	72	28	1
2. Teaches about professionalism.	47	42	11
3. Discusses his/her own strivings toward professionalism and his/her own shortcomings productively and sensitively.	34	53	13
4. Creates an environment of warmth and mutual respect in relating with students.	38	50	12
5. Is a good role model of professionalism for me to emulate.	41	43	16
6. Sets clear expectations for student’s professional behavior.	41	46	13
7. Enforces those expectation.	35	52	13
8. Explicitly describes the way a student should relate to patient in a difficult situation.	38	46	17
9. After describing the way a student should relate to a patient in a difficult situation, demonstrates that behavior for students.	32	44	25
10. After the demonstration, asks students what they saw and solicits their comments.	29	44	27

Table 6 shows the mean total scores of the medical students’ rating of faculty’s modeling and teaching behaviors in regard to professionalism for each of the four academic years. The mean total scores for all four years were approximately 58% of the maximum score of 30. The mean scores between each of the four years were not statistically different.

**Table 6:** Mean Total scores of participants’ rating of faculty’s modeling and teaching behaviors in regard to professionalism. Maximum score = 30.

Academic Year	Mean Overall Score
Year 2016–2017 n = 120	17.12 ± 4.24
Year 2017–2018 n = 135	17.62 ± 4.44
Year 2018–2019 n = 149	17.42 ± 4.08
Year 2019–2020 n = 116	17.51 ± 4.33
	***P*** **= 0.81**

## Discussion

Within the context of an Arabic institution in the Middle East, our study reports on medical students’ assessment of professional behaviors of their peers (medical students), residents and faculty, as well as their assessment of the teaching and modeling behaviors of the faculty. For all four academic years, the mean total Professional Behavior score was approximately 60% of the maximum possible score for all three targeted groups. This result is lower compared with similar surveys that reported on medical students’ or residents’ assessment of professional behaviors of peers or faculty. For example, in two studies conducted in Western universities, Arnold and colleagues and Quaintance and associates, using a similar survey, reported mean score percentages of 77.4 and 75.1%, respectively [15, 23]. In a study involving residents in a family medicine program at a Qatar university, Salem and associates showed that their mean total score percentage of professionalism was 70% [19]. Finally, Salem et.al, reported a mean score percentage of peer assessment of residents’ professionalism at an Egyptian university to be 71% [20]. While the latter two studies used questionnaires that differed from the one we used in this study, we measured similar professionalism constructs, which adds some credibility to comparing our scores to their scores.

Reasons that can explain the lower mean total Professional scores that we obtained at the AGU could include an excessive workload of students, residents, and faculty that could have an indirect negative effect on fostering professional behaviors. Workload and amount of direct involvement of the attendings in the clinical environment might have also limited the time for role-modeling. Finally, although AGU instituted a two-day workshop on professionalism for Year-5 students, a more intensive curriculum might be needed throughout all six years of the medical curriculum that also elicits the involvement of the faculty. This conclusion gains support from our finding showing that the ratings of the professional behaviors of the three groups remained unchanged during the four academic years despite the introduction of the workshop on professionalism. Qualitative studies are needed to further explore the reasons for these lower scores compared with other Arabic universities.

An interesting finding was that the medical students rated the professional behaviors of their peers, i.e., medical students, higher than those of residents and faculty in two of the academic years. While difficult to attach significance to these results (which may reflect positive bias towards their peers), it might suggest that the degree of professionalism declines as one passes through the academic ranks. In a study involving more than 10,000 students at Doctor of Osteopathic granting medical schools in the U.S., Hojat and colleagues observed a statistically significant decline in empathy scores when comparing students in the preclinical (years 1 and 2) and the clinical (years 3 and 4) phases of medical school (*P* < .001); however, the magnitude of the decline was small [24].

Additional evidence for a decline in empathy during medical training comes from the study performed by Baingana and associates, who conducted focus group discussions with 49 health professions undergraduate students (Years 1–5) from the 2008/2009 academic year at Makerere University College of Health Science [25]. Their results showing a variability in the way first- and fifth-year students conceptualized professionalism was emblematic of a loss of idealism. The authors hypothesized that the hidden and informal curricula had a negative impact on professionalism. In a qualitative study, Brown and colleagues showed that the hidden curriculum impacted negatively on professionalism and acted through role modeling, organizational culture, stereotyping and professional dress [26]. The students in the study also described their formal curriculum as being inadequate, as it consisted of one formal course that merely taught them how to “behave”. Students identified role models as being essential to the development of professionalism and their recommendations included the publication of norms of professional behaviors and the institution of training activities consisting of workshops and seminars.

Regarding the results of the subscales reflecting the different professionalism constructs, our results showed that for several of the academic years, medical students had higher mean scores for the domains “Honesty/Integrity” and “Accountability/Responsibility” compared with the other two groups. However, the mean score for the domain of “Honesty/Integrity” is lower than scores observed for a similar construct in the studies performed in Qatar and Egypt [19, 20].

In contrast, faculty had higher mean total scores for Respect/Caring/Compassion/Altruism compared with the other two junior groups. One could hypothesize that exhibiting professional behaviors related to “Respect/Caring/Compassion/Altruism” may require additional years of patient contact for doctors in training to achieve enhanced development of this professionalism construct.

In regard to faculty’s “teaching and modeling” behaviors, the mean total scores of medical students’ rating of their faculty’s teaching and modeling professionalism behaviors for all four years were shown to be only slightly more than half of the achievable maximum score, which is considerably lower than that observed in the study by Quaintance and colleagues, where the comparable score regarding student assessment of professionalism teaching was above 80% of the maximum [15]. The low scores given by the medical students at AGU regarding teaching behaviors of the faculty is mirrored by the findings obtained by Adkoli and colleagues [27]. These investigators used a qualitative approach consisting of 10 focus group discussions to elicit the views of final year medical students, interns, and residents to explore aspects of professionalism at the University of Dammam, Kingdom of Saudi Arabia. The participants at the University of Dammam considered very few teachers as serving as a positive role models and that professionalism was not taught or assessed.

Quaintance and associates hypothesized that “faculty who teach professionalism may behave more professionally” and in their study involving medical students, the students’ ratings of their faculty’s professionalism behaviors were positively correlated with their ratings of the faculty teaching professionalism [15]. Hence, there was a particularly strong relationship between the faculty teaching professionalism and exhibiting professionalism behaviors as perceived by the students [15]. In our study, we observed qualitatively and quantitatively that in general, the teaching of professionalism by the faculty was “mixed”, as rated by the students, which might have contributed to the students’ moderate ratings of the faculty demonstrating professional behaviors.

The structure of our study tool mirrored the a priori attributes of professionalism that include accountability, altruism, duty, excellence, honesty and integrity and respect, which are based on the ABIM domains influenced by Western culture. However, professionalism is culture-sensitive and any tool used to measure its constructs in the clinical situations should reflect the cultural context. To confirm the applicability of the tool we used in this study to the Arabic context, we refer to the study performed by Abdalla and colleagues who sought to determine the pre-clerkship medical students’ perceptions of medical professionalism. These investigators distributed an online survey to 300 medical students (years 1–3) attending the College of Medicine at University of Sharjah, United Arab Emirates (UAE) [28]. Participants were asked to describe an official doctor–patient encounter that they had experienced in a health-care setting and to highlight the professional behaviors in that encounter. The behaviors that the students mentioned covered all aspects of the ABIM Physicians Charter, which supports the appropriateness of applying the ABIM professionalism constructs to the Arab Region.

Two other studies showed the influence of the Arabic setting on medical professionalism constructs. One included the efforts of Al-Eraky and associates who aimed to formulate a professionalism framework for healthcare providers as interpreted by local medical professionals in Arabian countries [29]. These investigators recruited a purposive sample of 17 experts from diverse disciplines to participate in a Delphi study consisting of three rounds. Their panel validated the appropriateness of the six ABIM domains to the Arabian context but further proposed professional autonomy as an additional professionalism construct. Ho and colleagues investigated the applicability of the Western framework of professionalism to the local context in Qatar [30]. These investigators conducted 6 focus groups with 43 clinician-educators practicing at Hamad Medical Corporation in Doha, Qatar and while the participants generally expressed agreement with the applicability of the ABIM’s charter’s professionalism constructs to their context, they desired to enlarge the scope of patient autonomy to include family autonomy.

### Limitations

Our study had several limitations. First, one can theorize a selection bias in our study as the students who completed the questionnaire were those who attended the 2-day professionalism workshop and hence might have been more interested in professionalism than those who failed to attend. However, while attendance was optional for the first two academic years, it was mandatory for the latter two years and our results did not show any significant differences between the academic years. Another limitation was that the study relied on the subjective observations of the medical students, which might have been largely dependent on their memory. Finally, the demographical profile of the student body might limit its generalizability to other Arabic Universities. A major strength of this study is the inclusion of four different academic years cohorts in the analysis and the adaptation of a previous validated tool.

## Conclusions

This study provides valuable information to improve medical students’ experiences regarding the teaching of professionalism. Specifically, we will utilize our findings to a) discuss with the Faculty current approaches toward the teaching and modeling of Professionalism at the Arabian Gulf University College of Medicine and Medical Sciences and b) discuss with the faculty regarding implementation of changes in both the formal and informal curriculum throughout the basic science and clinical years to enhance professionalism teachings and behaviors. Our results also have policy implications on faculty recruitment, development, curriculum reform as well as further discussions regarding the influence of the organizational climate that supports professionalism.

Specifically, we recommend the following steps:

- Have the faculty perform a self-assessment of their teaching and demonstration of professionalism.
- Discuss curriculum reform and how professionalism can be further emphasized throughout medical school training.

## Supplementary Information

**Additional file 1.**

## Data Availability

The datasets used and analyzed during the current study are available from the corresponding author on reasonable request.
