# Supplementary material for: Perceptions of medical students towards the practice of professionalism at the Arabian Gulf University
Source: BMC Med Educ. 2021 Jan 8;21:38. doi: 10.1186/s12909-020-02464-z (PMC7792125; doi:10.1186/s12909-020-02464-z)
Supplement: Supplementary file 1 — Additional file 1. [file 12909_2020_2464_MOESM1_ESM.docx]

| **Climate of Professionalism Survey** | | | | | | | | | |
| --- | --- | --- | --- | --- | --- | --- | --- | --- | --- |
| **Directions:** Please rate the frequency that you have observed members in each group exhibiting each behavior during the past year. | | | | | | | | | |
|  | **Medical Students** | | | **Residents** | | | **Faculty** | | |
| **Professionalism Behaviors** | **Mostly** | **Sometimes** | **Rarely** | **Mostly** | **Sometimes** | **Rarely** | **Mostly** | **Sometimes** | **Rarely** |
| Show disrespect to patients, students, faculty, staff or other healthcare personnel |  |  |  |  |  |  |  |  |  |
| Advocate for the well-being of patients,  students, colleagues, the community and/or the medical profession |  |  |  |  |  |  |  |  |  |
| Make themselves look good at the expense of others |  |  |  |  |  |  |  |  |  |
| Exceed expectations in patient care, in the classroom, and at conferences and on rounds |  |  |  |  |  |  |  |  |  |
| Finish their work and help others finish their work |  |  |  |  |  |  |  |  |  |
| Complain about professional obligations |  |  |  |  |  |  |  |  |  |
| Does not reveal the whole truth to patients, professors, colleagues/peers or write false statements in the medical record |  |  |  |  |  |  |  |  |  |
| Show respect and compassion toward patients, students, faculty, staff or other healthcare personnel |  |  |  |  |  |  |  |  |  |
| At times, they hide their medical mistakes from their colleagues and the patients. |  |  |  |  |  |  |  |  |  |
| Ignore the unprofessional behavior of others |  |  |  |  |  |  |  |  |  |
| Do just enough to get by in patient care, class, conferences and/or rounds |  |  |  |  |  |  |  |  |  |

| **Climate of Professionalism Survey** | | | |
| --- | --- | --- | --- |
| **Directions:** Please rate the frequency that most appropriately describes how your faculty supervisor/attending physician taught and modeled professional behavior over the past year. | | | |
|  | **My Faculty Supervisor(s)** | | |
|  | **Mostly** | **Sometimes** | **Rarely** |
| Acts professionally in relating to patients, students, colleagues, and staff |  |  |  |
| Teaches about professionalism |  |  |  |
| Discusses his/her own strivings toward professionalism and his/her own shortcomings productively and sensitively |  |  |  |
| Creates an environment of warmth and mutual respect in relating with students |  |  |  |
| Is a good role model of professionalism for me to emulate |  |  |  |
| Sets clear expectations for students’ professional behavior |  |  |  |
| Enforces those expectations |  |  |  |
| Explicitly describes the way a student should relate to a patient in a difficult situation |  |  |  |
| After describing the way a student should relate to a patient in a difficult situation, demonstrates that behavior for students |  |  |  |
| After the demonstration, asks students what they saw and solicits their  comments |  |  |  |

Adapted from: Louise E Arnold PhD, George S Thompson MD and Jennifer Quaintance, PhD at

the University of Missouri-Kansas City School of Medicine
